# *Aspergillus fumigatus* spondylitis in an immunocompetent patient with annular high signal around the intervertebral disks: a case report and literature review

**DOI:** 10.3389/fmed.2024.1532282

**Published:** 2025-01-13

**Authors:** Zhihao Xu, Weijian Zhu, Sirui Zhou, Yuting Zhao, Qi Xiang, Yi Zhang

**Affiliations:** ^1^Department of Hepatobiliary Surgery, The First Affiliated Hospital of Jinan University, Guangzhou, China; ^2^Department of Orthopedics, Tongji Hospital, Tongji Medical College, Huazhong University of Science and Technology, Wuhan, China; ^3^Department of Respiratory, Li Yuan Hospital, Tongji Medical College, Huazhong University of Science and Technology, Wuhan, China; ^4^Department of Clinical Laboratory, KadWise Co, Wuhan, China; ^5^Department of Cardiology, Xie he Hospital, Tongji Medical College, Huazhong University of Science and Technology, Wuhan, China; ^6^Department of Orthopedics, Wuhan Tongji Aerospace City Hospital, Wuhan, China

**Keywords:** *Aspergillus fumigatus*, *Aspergillus*, infectious spondylitis, immunocompetent, clinical features

## Abstract

*Aspergillus fumigatus* spondylitis is a rare fungal infection, primarily occurring in immunocompromised patients, although cases in immunocompetent individuals have also been reported. While *Aspergillus fumigatus* is commonly associated with pulmonary infections, it can also cause spondylitis. Patients typically present with back pain, limb numbness, and neurological compression symptoms. Imaging findings often show vertebral destruction, reduced disk height, and paraspinal abscesses, potentially accompanied by characteristic ring-enhancing lesions. MRI findings can help distinguish *Aspergillus fumigatus* spondylitis from other conditions such as tuberculous spondylitis. This case involves an immunocompetent patient with *Aspergillus fumigatus* spondylitis, whose non-specific clinical manifestations can easily be confused with other types of spinal infections, leading to a potential misdiagnosis. Diagnosis requires tissue biopsy and microbiological culture. Voriconazole is the first-line antifungal agent, and studies have shown that it improves patient response and survival rates. For patients with significant spinal compression or neurological symptoms, surgical intervention combined with antifungal treatment should be considered if antifungal therapy alone is ineffective. Although *Aspergillus fumigatus* spondylitis is rare, it can occur in immunocompetent individuals. Early diagnosis through imaging and biopsy is crucial, and a combination of surgery and antifungal therapy can help improve prognosis.

## Introduction

*Aspergillus fumigatus* is a common opportunistic fungus typically associated with immunocompromised patients. However, spinal infections (*Aspergillus fumigatus* spondylitis) in immunocompetent individuals are extremely rare, making the diagnostic and therapeutic process challenging. The pathophysiology of fungal spinal infections typically involves the inhalation of fungal spores or hematogenous dissemination leading to damage to the bone structure. Particularly in immunocompetent patients, the mechanisms by which fungi invade the spine remain unclear and may be closely related to the local immune response of the patient, the fungal colonization ability, and other host factors (1). Most case reports in the literature focus on immunocompromised patients, so the documentation of cases in immunocompetent individuals is of significant clinical importance. Recent studies have reported several cases of *Aspergillus* spondylitis, with imaging findings showing high-signal annular changes around the intervertebral disks, underscoring the importance of early radiological evaluation (2, 3). This case report further demonstrates the rarity of the disease in immunocompetent patients.

## Case report

### Clinical findings

A 63-year-old male patient presented with over a month of lower back pain, which gradually progressed to bilateral lower limb pain and numbness. On clinical examination, the patient exhibited restricted lumbar spine mobility with tenderness on palpation of the paraspinal muscles from T12 to L3. Neurological examination revealed no abnormalities. Laboratory findings showed a WBC count of 5.01 × 10^9/L, CRP of 33 mg/L, ESR of 57 mm/hr, PCT of 0.03 ng/mL, and ferritin levels of 442.8 μg/L (Table 1). Tests for tuberculosis, including sputum culture and T-SPOT, were negative, as was screening for HIV.

**TABLE 1:** Laboratory test values at different stages of the patient.

Time	WBC (3.5–9.0 × 10^9)	N% (40–75%)	Lymphocyte% (20–50%)	CRP (0–10 mg/L)	A/G (1.0–2.5)	ESR (0.0–15.0 mm/hr)
Preoperative period	5.01	55.7	26.50	33	0.97	57
Postoperative day 1	7.77	75.1	16.2	69.5	1.03	52
Postoperative day 4	5.14	64.2	23.5	111.2	0.95	74
Postoperative day 7	5.62	65.4	30.1	54.8	0.88	62
Postoperative day 60	5.13	52.4	24.5	7.10	1.33	9

WBC, white blood cell; N, neutrophil; CRP, C-reactive protein; A/G, Albumin/Globulin; ESR, erythrocyte sedimentation rate. The value in parentheses is the normal reference range.

**FIGURE 1:**
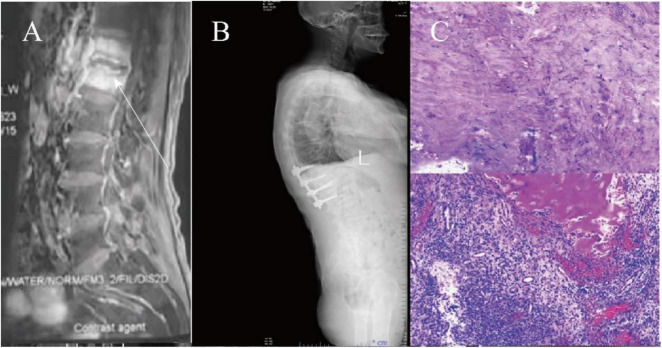
**(A)** Shows fluid sequence images. **(B)** Shows the postoperative plain film. **(C)** Shows the postoperative histopathological examination of the vertebral body and intervertebral disk. Necrosis and granulation tissue formation with inflammatory cell infiltration were seen in the diseased intervertebral disk and adjacent vertebral tissues.

Initial lumbar spine plain radiography performed at an external facility revealed narrowing of the T12-L1 intervertebral space and bony destruction along the adjacent vertebral margins. T1-weighted MRI sequences demonstrated localized low-signal intensity at T12-L1, with poorly visualized intervertebral disks and vertebral margins, alongside a localized defect at the upper margin of the L5 vertebral body. Fluid-sensitive sequences revealed diffuse high-signal intensity at T12-L1, reduced intervertebral disk height, preserved disk morphology, and a localized defect at the superior margin of L5. CT imaging confirmed narrowing of the T12-L1 intervertebral space, irregularities along the vertebral margins, multiple patchy hypodense foci, circumferential thickening of the paravertebral soft tissues, and hypodense lesions at the anterior-superior margin of L5 (Figure 1).

The 63-year-old male patient presents with chronic lower back pain and bilateral lower limb symptoms. From an immunological standpoint, the patient’s white blood cell count (5.01 × 10^9/L) is within normal range, and procalcitonin (PCT) is low (0.03 ng/mL), indicating no acute bacterial infection. However, elevated C-reactive protein (CRP, 33 mg/L), erythrocyte sedimentation rate (ESR, 57 mm/hr), and ferritin (442.8 μg/L) suggest persistent systemic inflammation, possibly linked to chronic infection or immune dysfunction. Tuberculosis and HIV screening results were negative, ruling out major immunosuppressive conditions. Although immune cell counts are normal, the elevated inflammatory markers and clinical presentation raise concerns about potential immune dysfunction, which may affect the patient’s response to infections, including fungal infections. Based on the immune and laboratory results, while no clear immunodeficiency is evident, the patient’s chronic inflammatory response requires attention, as such patients may present with different clinical manifestations and treatment responses when faced with fungal infections.

### Therapeutic interventions and follow-up

After obtaining the patient’s informed consent, a CT-guided posterior percutaneous vertebral biopsy was performed to further investigate the underlying cause (see Supplementary File 1). The biopsy samples were subjected to bacterial culture and drug sensitivity testing, which yielded negative results. Based on these findings, the patient was subsequently started on empirical antibiotic therapy, including ceftriaxone 2 g twice daily (BID) and cefoperazone sodium 3 g once daily (QD). However, the patient’s back pain and neurological symptoms did not show significant improvement. Imaging studies revealed high-signal annular changes around the intervertebral disks. Considering the clinical presentation, we continued to favor a diagnosis of fungal spondylitis. To promptly and effectively relieve the patient’s symptoms and improve their condition, the patient underwent posterior lumbar lesion excision, autologous iliac bone grafting, and pedicle screw fixation, after obtaining full informed consent, to restore spinal stability and clear the lesion (see Figure 1). Postoperative fungal tissue culture and Next-Generation Sequencing (NGS) identified *Aspergillus fumigatus*, while *Mycobacterium tuberculosis* cultures were negative. Consequently, the treatment regimen was switched to Voriconazole. The initial dose was 400 mg IV for 3 days, followed by 200 mg IV every 12 h for 5 days, after which the patient transitioned to 200 mg orally twice daily. A 1-month course of outpatient antifungal therapy was recommended. Despite treatment, the patient returned 2 months later with persistent low back pain. Follow-up lumbar spine MRI revealed post-T11-L2 internal fixation changes, with abnormal morphology of the L4-L5 vertebrae, showing patchy long T1 and T2 signals in the vertebral bodies and faint long T1 and T2 signals in the adjacent compressed soft tissues (Figure 1). Voriconazole was reinitiated at 200 mg IV every 12 h, and the patient showed symptomatic improvement after another month of treatment, leading to discharge.

## Discussion

*Aspergillus* spondylitis clinically manifests mainly as back pain and can cause numbness and pain in the limbs and other nerve compression symptoms. Its clinical manifestations are not specific. On lumbar spine MRI, vertebral body destruction, decreased disk height, and paravertebral abscess are mostly seen, and the vertebral body is the most common site of infection (4). Furthermore, *Aspergillus* spondylitis shows an annular high signal around the intervertebral disks on fluid sequences (Figure 1), which may be a more specific imaging feature of *Aspergillus* spondylitis. This sign is specific in infectious spondylitis, where pyogenic spondylitis tends to show intervertebral disk destruction with diffuse high signal in the vertebral body on the fluid sequences, and tuberculous spondylitis tends to show small intrabody abscesses, sublimated diffusion of the vertebral body in the anterior part of the vertebral body, and gross destruction of the vertebral body (4). Unfortunately, this sign has not been specifically reported in previous reports, but similar signs have been observed (5–7). However, paravertebral abscesses involving multiple vertebrae in a row can be easily confused with tuberculous spondylitis, making the diagnosis challenging (5–7). At the same time, this sign warrants a large-scale search for cases for a controlled clinical study, intending to differentiate it from tuberculosis better and increase clinicians’ attention to *Aspergillus* infections. Although the probability of *Aspergillus* spondylitis occurring in immunocompetent individuals is extremely low, an annular high signal around the intervertebral disks on fluid sequences should be a cause for concern and supplemented with antibiotic therapy to further the diagnosis. Early diagnosis can help to alleviate the patient’s pain and reduce the symptoms of nerve compression to improve the long-term prognosis (4).

The most reliable diagnostic methods for *Aspergillus* spondylitis are histopathologic examination and tissue bacterial culture (8). When inflammatory indicators and imaging tests suggest *Aspergillus* spondylitis, puncture biopsy with empirical antibiotic therapy should be performed as early as possible. In addition, blood cultures should be actively performed, and although the positive rate of blood cultures is low, the positive results can still guide the choice of antibiotics. According to the latest guidelines for managing *Aspergillus*, Voriconazole is recommended as the optimal treatment for *Aspergillus* spondylitis (9). The study showed that using Voriconazole in patients with invasive *Aspergillus* infection was associated with better response and survival, leading to less serious side effects (10, 11). Conservative antifungal therapy is ineffective when there is epidural abscess formation at the site of spinal infection, spinal cord compression, spinal instability, and the onset of neurologic symptoms. At this point, surgical combination antifungal therapy should be considered.

However, despite these advances in treatment, there remains a significant gap in the literature regarding the optimal management strategies for *Aspergillus* spondylitis, particularly in immunocompetent patients (Table 2). Further investigation into the role of combined surgical and antifungal therapy in cases with spinal cord compression or epidural abscesses is urgently needed. Moreover, while the pathogenesis of *Aspergillus* spondylitis is still not fully understood, it is likely that the infection may arise from either direct inoculation following trauma or hematogenous spread from distant sites of infection (3). This suggests that *Aspergillus* species may exploit specific host vulnerabilities, such as impaired immune responses, making it critical to identify common risk factors such as diabetes, malignancy, or recent surgical interventions, which may predispose individuals to fungal infections.

**TABLE 2:** Clinical characteristics of *Aspergillus* spondylitis (AS) in immunocompetent (IC) patients.

References	Year	Sex/age	Predisposing conditions	Presentation	Radiology	Diagnosis	Species	Therapy/duration	Outcome
(5)	2003	63/M	Diabetes mellitus	Fever Tetraparesis	MRI (C2–C5)	Surgery	*A. flavus*	Laminectomy Itr	Meningitis Death 2 weeks later
(6)	2003	52/M	Past pulmonary tuberculosis	Paraparesis	MRI (L2–L3)	Biopsy	*A. fumigatus*	Laminectomy L-amB, Vor/6 months	Recovered after relapse
(7)	2004	48/M	IVDU		MRI (T7)	Biopsy	*A. fumigatus*	Itr/18 months	Recovered
(12)	2004	35/W	None	Paraplegia	MRI (T10– T12)	Surgery ELISA Negative IEAS	*Aspergillus* spp.	Corpectomy AmB–Itr	Drug toxicity and death 2 months later
(13)	2004	50/W	Discectomy	Sciatica	MRI (L4–L5)	Surgery	*A. fumigatus*	Itr/3 months	Recovered
(14)	2007	51/W	Discography ESI, COPD		MRI (L4–L5)	Biopsy	*A. fumigatus*	Caf. Vor/5 months	Meningitis and death 5 months later
(15)	2008	43/M	PA		MRI (T6–T9)	IEAS Biopsy	*A. fumigatus*	Vor/5 weeks	Recovered
(16)	2009	50/M	PA Diabetes mellitus	Paraplegia	MRI (T2–T8)	Surgery	*A. fumigatus*	Laminectomy Vor	Death 2 weeks later
(17)	2010	66/W	Diabetes mellitus	Fever Paraparesis	CT (L4–S1)	Biopsy	*Aspergillus* spp.	Laminectomy Vor	Recovered
(18)	2010	53/W	None	Fever Paraparesis	MRI (L2–L3)	Biopsy	*A. fumigatus*	AmB	Death 2 months later
(19)	2011	52/W	PA		MRI (L2–L5)	Surgery	*A. fumigatus*	Laminectomy Vor/7 months	Recovered
(20)	2013	65/M	Diabetes mellitus	Headache Cervical pain	MRI (C2–C3)	Biopsy ELISA/IEAS	*A. flavus*	Vor/12 months	Recovered
(21)	2013	40/W	Lung fungal Granuloma brain cysticercosis	Back pain, numbness weakness	MRI (T1–T3)	Surgery Histopathological	*A. nidulans*	Laminectomy Vor/6 months	Recovered
(22)	2013	33/W	Spinal Anesthesia	Back pain	MRI (L2–L3)	Surgery Histopathological	*A. fumigatus*	Discectomy Vor/11 weeks	Recovered
(23)	2013	45/W	Diabetic mellitus	Back pain	MRI (L5–S1)	Surgery Histopathological	*A. fumigatus*	Laminectomy Itr/3 months	Recovered
(24)	2014	53/M	Spinal block procedures	Motor Weakness Paresthesia	MRI (L2–L3)	Surgery Biopsy	*Aspergillus* spp.	Laminectomy AmB/30 days	Recovered
(25)	2015	20/M	Past pulmonary tuberculosis	Fever Back pain	MRI (T7–T12)	Biopsy Histopathological	*A. terreus*	Vor/16 days Caf/40 days	Sequelae
(26)	2019	61/M	Lumbar steroid injection	Back pain	MRI (L3–L4)	Surgery Biopsy Histopathological	*A. nidulans*	Lumbar discectomy Caf/6 weeks Vor/7 months Pos	Recovered
(27)	2020	43/M	Brucellosis-related vertebral	Back pain	MRI (L5–S1)	Biopsy ELISA	*Aspergillus* spp.	Vor/3 months	Recovered
(28)	2015	53/M	pulmonary granulomatous	Fever Cough Back pain	Chest CT MRI (L2–L5)	Surgery	*Aspergillus* spp	Vor/3 months	Recovered
(29)	2019	71/M	abdominal stab wound	Back pain Paresthesia	MRI (T11–T12)	Biopsy ELISA Histopathological	*A. terreus*	Laminectomy Vor/5 months	Recovered
(30)	2022	54/W	None	Back pain	MRI (T11-T12)	Surgery Histopathological	*Aspergillus* spp	Laminectomy Vor/3 months	Recovered
(31)	2023	68/M	None	Thoracolumbar back pain	MRI (T12–L2)	Surgery Histopathological	*A. fumigatus*	Isa/12 months	Recovered
Our case	2024	63/M	None	Back pain	MRI (T12–L2)	Surgery Histopathological	*A.fumigatus*	Vor/10 Weeks	Recovered

PA, pulmonary aspergilloma; COPD, chronic obstructive pulmonary disease; ESI, epidural steroid injections; IVDU, intravenous drug user; CT, computed tomography; MRI, magnetic resonance imaging; IEAS, immunoelectrophoretic analysis of serum; AmB, amphotericin B; Caf, caspofungin; Itr, itraconazole; Vor, voriconazole; Isa, isavuconazole; Pos, posaconazole.

In conclusion, reporting cases of *Aspergillus* spondylitis is of paramount importance to raise awareness among clinicians, particularly in atypical cases or those involving immunocompetent patients. The case we present highlights the need for further research into the pathogenesis, diagnostic markers, and treatment strategies for *Aspergillus* spondylitis. Establishing more robust clinical guidelines will improve early recognition, treatment outcomes, and overall prognosis for patients affected by this rare but serious infection.

## Conclusion

*Aspergillus fumigatus* spondylitis also needs to be considered as a possibility of mycobacterial spondylitis in the presence of normal immune function and a history of no risk factors, and prompt diagnosis and treatment can help improve the patient’s prognosis.

## Data Availability

The datasets presented in this study can be found in online repositories. The names of the repository/repositories and accession number(s) can be found in this article/Supplementary material.
