# Corrigendum: *Aspergillus fumigatus* spondylitis in an immunocompetent patient with annular high signal around the intervertebral disks: a case report and literature review

**DOI:** 10.3389/fmed.2025.1593828

**Published:** 2025-03-26

**Authors:** Zhihao Xu, Weijian Zhu, Sirui Zhou, Yuting Zhao, Qi Xiang, Yi Zhang

**Affiliations:** ^1^Department of Hepatobiliary Surgery, The First Affiliated Hospital of Jinan University, Guangzhou, China; ^2^Department of Orthopedics, Tongji Hospital, Tongji Medical College, Huazhong University of Science and Technology, Wuhan, China; ^3^Department of Respiratory, Li Yuan Hospital, Tongji Medical College, Huazhong University of Science and Technology, Wuhan, China; ^4^Department of Clinical Laboratory, KadWise Co, Wuhan, China; ^5^Department of Cardiology, Xie he Hospital, Tongji Medical College, Huazhong University of Science and Technology, Wuhan, China; ^6^Department of Orthopedics, Wuhan Tongji Aerospace City Hospital, Wuhan, China

**Keywords:** *Aspergillus fumigatus*, *Aspergillus*, infectious spondylitis, immunocompetent, clinical features

In the published article, there was an error regarding the affiliation for Yi Zhang. Instead of affiliation 1, Yi Zhang's affiliation should be affiliation 6 “Department of Orthopedics, Wuhan Tongji Aerospace City Hospital, Wuhan, China”.

The authors apologize for this error and state that this does not change the scientific conclusions of the article in any way. The original article has been updated.

