# Supplementary material for: Aspergillus fumigatus spondylitis in an immunocompetent patient with annular high signal around the intervertebral disks: a case report and literature review
Source: Front Med (Lausanne). 2025 Jan 13;11:1532282. doi: 10.3389/fmed.2024.1532282 (PMC11771204; doi:10.3389/fmed.2024.1532282)
Supplement: Supplementary file 1 [file Data_Sheet_1.docx]

>V350057461L1C005R0100571834

AAACTAATTTAGAACTATAATACTATTAGTTATAGTTCCTAAG

>V350057461L1C004R0050504222

GCCCGGATTTGAGTCGGGCCTAAGTGGAGCCAAACAATGGCCG

>V350057461L1C001R0151308863

GAGGATTGGGGGGTTTGTGAGCCAATACCACGGGCGATGTGAG

>V350057461L2C003R0400966880

TGTATAACTTACTATACCATGAATACCAGTGCTGATAGAAAAT

>V350057461L2C006R0350201991

GTGCGATATTTGGTGCTCCAACCAGAGTAGACGACCCGAAGTG

>V350057461L2C004R0181175065

CCCCACCCGACCGACTGATTTTCAGATCCGCTCAGTACATAAC

>V350057461L2C003R0260501331

AAAGGGTTGTCCTATTCTTTGGAGAGCTACATCTCTCTTTTCG

>V350057461L1C006R0320072095

GGATTTAATGGAAACGCCTGAAACACTGTATTGTTGATTATAG

>V350057461L2C005R0020252009

GAGAGAGCCTGCGAGAGAGAAGATGGATCCGAATGATTTGACT

>V350057461L1C003R0041024657

AAAAAACTGAATTACAGGAAACATATACCCCTGGCGACGTGCG

>V350057461L1C006R0280621365

CCCCGCACTCCGGTCCCACAGCCAATCATCAGCAGCAATCCGG

>V350057461L1C006R0061352836

AGTGACACCTTCTGGCGATAGGAATCTGCTCCAAGAATTATAC

>V350057461L2C002R0280679488

TTCTGGACCGAACCCCAGAGCTTGCTTATCGCACTGATGCAGC

>V350057461L1C006R0160404016

CACGGTAAACACCCTTGTACGGAGTAATACTGGATGTACCGGC

>V350057461L2C002R0060990649

GAAGCGAATTTCGGGCCCCTTGGGGAGAATCGACCCGATATCG

>V350057461L1C004R0541111137

ACCTAGTATCAGAGTTCCAGCCTGTACGGCTTGTGTTTCTCAG

>V350057461L2C001R0570661215

CCTCAATGCCCGGACGGATTCCCACCCGTGGGCGACAAGTGCC

>V350057461L1C006R0110849509

CGTAGTGTCAGTCCGTGGGTGATCAAGGACAGGTCACATCCAC

>V350057461L1C002R0020874258

GATCGAACGAGGAGTTGCTCCCACTGTCGAGATGGGTGCCACT

>V350057461L1C003R0200808756

TTCGAAAATTAGAGAACTGATGATAGCCTCATCAACGAGTCCA

>V350057461L1C003R0261038525

CTGCATTTATTCTTCTATTCCATCTACTCAGCCTTGGTTCCAG

>V350057461L1C002R0201156791

CAGTGATCTGCTGCTCGGAGCAGTCGACGTCAATGCGCCCAGT

>V350057461L1C001R0560442626

CTGCTACTCTTCTGTGGACTAGCTGTGGATGCTGCTCTGAGAC

>V350057461L2C006R0110644299

GTGGCCGTGGCCGGTATCAGTGGGAACACCATCCTCAAAGGCG

>V350057461L2C006R0151101539

GTCAAGAGATGGTATATCCTACTCGCGTCCTCTAGGCTCTCAA

>V350057461L1C001R0600476158

GNTGAATACTGCCCTACTGCTAGCAACAGACACGATCTTACGG

>V350057461L2C004R0640409684

TCTAGGTAGCAATCCATAAGCACCATAGAAGGACTCCAATAAA

>V350057461L2C002R0280287164

TTCCGAATTTACAGGCGTTGGTTCCAGACAAGGGAACTGTATC

>V350057461L2C005R0520480954

CAGTATTGGAAATGCAGCCACACGATCGGTGGCGGGAAGAGGT

>V350057461L2C005R0560270268

TGGAGAGGGTGATCAAGCCGAATAAGGATAATATCTATGTGCT

>V350057461L2C002R0220154331

ATCGAGGTCATTCCCAGGGCACCCGCCAGGGAATCGGTTGCCT

>V350057461L1C004R0161123134

ATACTGACCAAGCTCATGGGAAACCAACCTATGATCCACGCCC

>V350057461L1C004R0321057568

CAGTTGGAGATCCTAGAGCCGAATCTCCCATTGTTATCAGAAG

>V350057461L2C002R0390683917

CACGAAGCGACATCTCTCCTCCAGCGGAACATAGTAGCCACCG

>V350057461L1C004R0641277264

CCAACCTGCACTGTGCCAAGCCAATTCAGCGTGCCGTCCGCAT

>V350057461L1C002R0020325077

GATTACCTCCCCGGCCCTGAAGACAGTCTAACCCCCAAGATAG

>V350057461L1C001R0311143693

ATCGCGTCTGTGTGATGGGTTAGAGAAACAGACCTCTTCTCCG

>V350057461L1C003R0191354517

CAATTGAAAAGGTATTGTCGTATTGCCGAGAGTCTGATATGAT

>V350057461L1C006R0070237716

GCAGGACAACCGCATATTTCGGATCACGTAGGTACTTTGGAAC

>V350057461L1C006R0241312640

CCGGGTGACACTGTCCACAGCGAATCTATGACTGCGCCGTAGC

>V350057461L2C004R0130742466

ATAGGGATTGATATCCAATCGGCTTCGTGAATGTTGGTATTAA

>V350057461L1C001R0490385502

AGTGCTGTTATCTCCAGGACAATTACATTAACGACTACCTATC

>V350057461L2C006R0540579009

TCGGATACTCCTGTTCACAAGCTGTCCTACCCCGAACGCGAGG

>V350057461L1C005R0220815682

TTCCTCTCAGATTCTGCTACCGGGGAAATAGGGTCTTTTCATT

>V350057461L2C002R0651335842

CTTGGACCGCTTCATATTCGCCTCGTCGATCATAGATCGCCCA

>V350057461L2C002R0270233648

CGGCGTATATCACCCATGCTTCCGGATTTACCACACAGGTGAC

>V350057461L2C003R0230407827

CTTTTATTAATAGTCCTAGAGACAGAGGTTAATTAAAATATCT

>V350057461L1C005R0720157836

CCCTATGCAAGCAAGATTAGCTGCAGATTTCAAGGGGTGCCTG

>V350057461L1C004R0570028279

ATCCATACTGTTTTCCGAAACCCCACAAATGTCAAGCAAACGA

>V350057461L2C002R0550785964

CTAGGACCAGTGAACGAAATTGTGACTCCCATGTCTGTTATCT

>V350057461L2C001R0540491031

TTATCCACCTCATCAACATCGAGCTCCTAGCAGCGGATTCAAC

>V350057461L2C004R0220679999

AGGATACCTACCTATGACAGACTAGCTGGTTAGCCATTGTGCA

>V350057461L1C001R0300312765

TTCTACTACCACCTTCTCATAAAGAGAAGGCTAGAGAGGGTAT

>V350057461L1C002R0641241235

TGAATTCACTGCTCTGAGGGAGAGATGTCTGAGACAAAGGAAG

>V350057461L2C001R0651118466

TGATTCTCGAGCGTGGAGAGGTTTGAAACATACCCATAGTGAC

>V350057461L1C005R0300435678

AACAGAGCCGAGTCCCGCAACAGACCCCACATTTAAACCAAGA

>V350057461L2C001R0640257252

CTCCTGACCGTTTTGGTGGCGGTTATGCAAATTTGAATCAGGC

>V350057461L2C001R0530878934

ACCCCATTTCTCTGAGCCCGTTGCAAGGAACCCAAGCATTACG

>V350057461L1C005R0670620851

GTCATTGACTACACACTTAATGCCACTGCGCGTACATTTGGTG

>V350057461L1C003R0130597743

AGTTCTTAACTGGGTCAGCTGCTTGATCTAGTGGTACCGGAAT

>V350057461L1C006R0081117700

AAGTCGGTTTTGATCTACGAGATAATGAGAAACTAGTATACGG

>V350057461L2C006R0410623600

CGATCTTAACGACGAATGAGATACTACAACACGAAAGTTTGTC

>V350057461L2C003R0111285877

GTGGAAGGCCTTGAATCATCTCGCTCCTCTATCGTTGCAAACC

>V350057461L1C003R0510032820

TACGTCTATGCTTGACGATGCCACGATACATGGATGCCCGAGC

>V350057461L2C004R0531195420

TCAGCGTTTGTATTACTATGAACTCCAAGCCGGGTAATTCTCT

>V350057461L2C003R0240895116

TCCCATGGATTATAGAATGAGCGTGAGCATGCAATACGATGGC

>V350057461L1C004R0640731006

ATACTCTATCGTCGTCCTGTAGGTGTAATTTCCTTCAAAGCCG

>V350057461L2C002R0310387128

CTTGACATCAAGAGGGCAGAAGACCTCTCCTCAGTAGAGCTCC

>V350057461L2C003R0580453423

CGATCGCTCTCCGTGTCCTGTGGCGAAGTTTCTGCGCTGTTCG

>V350057461L1C003R0720917941

GGATAGAACCTTGTCCCCAACATGTTCTCGAACTACTCAGTCG

>V350057461L2C001R0720582402

ATGGATGAAGGCGTGTTTCAAAATTTTAATAAGCAAAACCATC

>V350057461L1C004R0680411525

AGCGCAGAGGCCGCATTTCCGTCGATCATGCGGTCGATATTCT

>V350057461L1C002R0300334575

GTCTGAGAACCCAGGTGACTGCTGTATGCTCAATATGCTAACT

>V350057461L1C002R0470251949

CACAGATCAGGTGATTATTTAAATAACGATTTAAGGAGTTGCC

>V350057461L2C006R0050443312

TACTCTTCTGGGAATGTGGCGAGACCATCAAGGAGTCGAGTGA

>V350057461L2C003R0410751432

ATGACCGCAATTTGAGAAGAAGGACGTGAGTAACTGACCTTTG

>V350057461L1C005R0200771844

GGAAAGCAGCTGATCGTTAACGGTCGTCCGTTTCTGATACTGG

>V350057461L1C003R0310640063

CCCACTAAGCAACGCCACGATGCCCAATGGAAAGAGCTCGAAG

>V350057461L2C001R0301192581

TATATCTCAGTAATGCTAATACAGGTCTTGAACTGTCCCAACA

>V350057461L1C001R0580631803

CATTCGGTGACCCTCTATAGCTACGGCACTCCGAGGGCTCAAC

>V350057461L1C006R0310510018

GGTTCTCGGATGCTTTCCGCTGCTGCTTAGTCCAGAGTTTTAC

>V350057461L1C002R0411047801

AATTCTTGATTTCCTGCAGAATTCAAACCAAGCTGACTTCATA

>V350057461L1C003R0130171516

AGATAAATAGGTTGCTAAGCAGCTATTTCTGGCCTAGATACTC

>V350057461L1C006R0501095082

ACAGCAATCGCTGTTGCTTCGTCTTCGGAGATGGGACTGTTCT

>V350057461L1C001R0440458277

GTAGCACAACATTAACACACGCAGAATTCGTCATGACATCCAG

>V350057461L1C004R0190700566

CTCCGACCTCGCTTCGATGTTATCCCGGTCCCGAAAGCCTTTT

>V350057461L1C002R0330849827

GGCATTGGCATCTGGGTCCCGGGCTGTGTTCAGAAAAGCTGTT

>V350057461L1C001R0031103120

TCCATGCTCTATTGGCCAATGAACATGGTCATGCCTAAGGCAG

>V350057461L1C004R0110624169

CACCTGCTGTACTCCACGTGCGCTTCACATGACTAGTGCTAAT

>V350057461L2C004R0610918640

CCGTAATCCTATGTACATCAACAACGGTCTCGTGTTTCCCAAT

>V350057461L2C005R0311003484

GTAATGCTTGACAGCTCCTCGCGAAGCTTTTGATCGAGCTGAC

>V350057461L2C002R0640482952

GAAGCGGGCGTGCGATGCGCCTGCTCGTAGAGACCGGCACGCG

>V350057461L2C004R0150469911

ATTGGCTGCAGGTCTCCTTCCCATTGGGCGATGATGTCTTCCA

>V350057461L2C001R0170417720

GACTGAATTACTTGCTTCCCCTCCCTATATGCGTCAAACACAT

>V350057461L1C004R0681224464

CCAACTTTCTGCCGCCATCTGCCGAGAACCGTTAGCAAGTCTG

>V350057461L1C001R0180761891

ATCCATTTTCGTTTGAACTTACTTCTCTACATCCCCGAATCTC

>V350057461L2C005R0260107003

CTCGGAGCGTACTCTCATCTACGATATGACGTGGACGAGTACA

>V350057461L1C006R0381159408

CTCGTACATAACGGTAGCTCCATGATGTCATTATCACCAGAAC

>V350057461L1C003R0530337255

GTGAAAGGAAACCCGAAGACGAACTGAGTTCAGACTGATTCAT

>V350057461L2C006R0191225819

TTATTATAGTTAGTATTTTCTAAGTCAGGACTGATAACCTGAC

>V350057461L2C004R0310764739

CAGTGACTATGTGTGGTACCTTCCTCCCAGGCTTTCTGGACCA

>V350057461L2C003R0240908253

CCTCGGTGACGGAGCTCTGGGCTCCAAAATATGAAGTGAAAGC

>V350057461L1C002R0270532539

TTTGCCATCCATTCTATGGTAGGAATAACACATAATGTACAGG

>V350057461L1C006R0150860730

ATCATCCATCATGGCTCAATTTCATCCCACTATTACCCCGGAG

>V350057461L2C006R0500829689

CGTACGTTTGCTCCGGCGATGTCGCTGAGAATGCGGGATATCA

>V350057461L2C002R0670164176

TATTCAATAATTACACCTACTCTTGCACTAGAACTAGAGAATA

>V350057461L1C006R0070943754

AACCTAGTCAAGGAGGGCTTACCCCCCGGAGCACGGAAGCGGA

>V350057461L1C006R0190027313

CCCCACTCGCGGAAGCGGAAGTTGATGGCGAACCGGCGGGAGC

>V350057461L2C002R0140096384

CGTTCAGAATCGCGCCGCTCCCGGTCTGGATTCCGCAGCCAAG

>V350057461L1C004R0460479911

CTGAGAAAGAGATACACAAACGCAGGAATAAATGGCACTCCAT

>V350057461L1C004R0461352797

ACTTGCGGTTCACAAGCCCGTCAATACTGGGGCTATATGTGAG

>V350057461L1C006R0350351310

TTCCAAGGGAAATGACATACCTGAAGACCATATCCGTGTTCAG

>V350057461L2C002R0440932659

GGTGTTACTATCTATGGTGCGAGGGTGAAGAAGCTGTGCAAGC

>V350057461L2C005R0420964157

AAACAAAATTTCTCCGTTTATTGTACAGCTGACATTGCCGAAC

>V350057461L2C004R0670808114

GTTCCGGCGTCGGACTTGAAAATGTCCATCTTACGCTACGATC

>V350057461L1C001R0171061263

GTTAACTCTTGCTTCGTACCTCTGGCGTACAGGCAATTGAACT

>V350057461L1C005R0040416737

CCTTCATCCATCTTTTGGGACCGGCAGAAGAAGCATTGACTAA

>V350057461L1C006R0480153065

ATACCATGAGGGATGTAAGATTCCCTGTCCCTGCCTCTTTCCA

>V350057461L2C006R0360732438

CCAGAAGATCGTTCAGAGCATGTCACCATTACCCTACTTACTG

>V350057461L2C005R0670775249

TGATCAGCAGGGGTCTCCAAGCTCCCCCCAGACTCGCAACCAC

>V350057461L2C003R0280846151

GTGATATAGTTCGTTATACGCTGGAGAATGACTCAGGCCAAGA

>V350057461L1C001R0680270139

ATGGTACTGCTGTGGATAGGAGTTGGTATTACTAAGAAGGAAT

>V350057461L1C006R0380557062

AACATGTCTTTGCAGAAAAACCATAGGGAAATATATGTTTCCA

>V350057461L1C006R0161291128

CCTTGGTAGGTTTCTGAACCCCGCTGCCGGACATGGCTTTTTC

>V350057461L1C006R0710126617

GAATTACAACACAACGGATCAGCTCTGACAGAACAGGTCTCCT

>V350057461L2C001R0190597811

GAGCCCGGCGGAATGATCAGCGTGTCCTTGACGATGTACACAC

>V350057461L1C005R0131179554

CAGTATTGCGAACCAGAATGGGGCCTTCCTAAGCGATTGCCAT

>V350057461L2C001R0030643589

AGAATACTACCATTAACTAAAAGGGTGTCCGGGCGATTCCTAT

>V350057461L1C004R0200909868

CTCAACCCGCGTTGCGAGCCAGGTCGGGTTTGACTTTCTGTAC

>V350057461L2C005R0660442118

TCGGGATCCCCGTACTGGCTTAGTAGTGTACGGGGTTAGGGAG

>V350057461L2C006R0420079363

AATGACTCCAAGACGCCTAGCAAGACTTATCTGCTTATCACAA

>V350057461L2C005R0690189802

GGGAGATGAGGCCCGGTAGGCCCAGTAGGCCCAGTAGTGCTAT

>V350057461L1C002R0220563314

CTCTACGATGGAACAATTGCTGCAGATATTCCTCTTTCGGAAG

>V350057461L2C005R0280866836

TCTCGACTGACGCTTTATAGCTTCAGCAGCAGGCTGGAGATCA

>V350057461L1C002R0670672081

GTTTTGTCCTGTGTTCCATCATGTCTTTCCAGTTATATGATGG

>V350057461L1C001R0401044030

TGAATGAATGACATTGCCCACAATGGATCGGAATTGACCTCAA

>V350057461L1C005R0541058083

TGCCTTTTATTACCACCTGAGACATTTATCAAGTGCAGCGTTG

>V350057461L2C003R0501251254

AAGCACTCAACCATAAGAAACCACTCCGTGTGTCAGAATGAGT

>V350057461L1C002R0400212568

GCAGCTAAGACGCAGGCGACTACTCCTCAGTGTTTCTTTACCA

>V350057461L1C003R0091255260

GGGCGTCAAAGCCTCGATGGAATATCATGCTTACAATTACCTA

>V350057461L2C006R0050210352

GTTTTGATATCCATAGACCATAGTATGGAGTATGCTGTGTATA

>V350057461L2C001R0360193454

ATTTCGATGTTGACTATCAGTTAATCTACAGTCGAAAAAAGGA

>V350057461L1C004R0250143717

TAGTGAGTTGAGCGATAGACAAGCGCTAATGACTTCGACATAA

>V350057461L1C006R0080185481

TACGTAGTGAAAATGACGTGGTCAGGTTTCACTTTGTTCTTGA

>V350057461L2C003R0360378524

GCAGTCTTCGCTGCTAGCAAGCATGCTGCAGTCGGGATGGTCA

>V350057461L2C006R0191220326

GGAAGAGAGTGACGATCTGCCTTCGCTGGAGAAGACGCTTGAC

>V350057461L2C003R0620171631

GGCCTGTTATCATCTACGGCGCGTCCACGGCAATTGGCGCATT

>V350057461L2C003R0410423837

GATTGCAACTGATTTGATTTTGCGAGGCGTCAGTGACGGTTTT

>V350057461L1C004R0181017756

AGAAAGAGAGGCTATTGTGTGAACTTGTTAGCACGTGCTCATG

>V350057461L1C001R0510255463

TCACGAATATAGAGGGGAATAGATGGCTTGCCTAGGTATAGAG

>V350057461L2C005R0200469641

TGTTCTCACTATATGGCTTCATCTCAAGTCTATGAAGATTCAG

>V350057461L1C002R0640084318

AATCTCGGGTTGAATTTAGCAGTGCTACCTTGAGGAATGTTAC

>V350057461L2C003R0420257176

CGCCTCATCGAGACATCCCGCAAAGTGCAGGACAACATCGTTG

>V350057461L2C004R0091195639

GACACTATCAATAACCTTAACAACTCGACTCACTATCTCGTCC

>V350057461L2C003R0100028215

CGCCGGCCCACAGGGCCGAGCAGGCTGCCTGCACTGCCGCGAG

>V350057461L2C001R0470805635

GTCAGGATAGGCCGTATTCAAGAGATCCACATAACGATATCCG

>V350057461L1C001R0261123595

GCCGGCCCATGGAACCAAACCAACCTCTTGCAGTCAACCTAGC

>V350057461L2C006R0271167956

CACACAGCATCACAGCATTGCCAGTCCGCTGGGCACTTGTCAC

>V350057461L2C005R0071058071

CAGATCTGCTCGGTCATCACAAATGGTACTGCTGCTGTCCCAT

>V350057461L2C002R0640617303

ATTCATTTGATAAACCCATCTGAGCTGAAGAAATGTGCTAAAT

>V350057461L2C004R0360292152

ATCCCAGTACACGCCTAGTAACACTACAGCTTGGAGGACTGTG

>V350057461L1C005R0040266870

CTGTAATTTAAAGATGCTATTTATATACCTACTAGTACTACAG

>V350057461L2C006R0021168296

CATGTAACATATGCCCTTTTCCCTGTCTGTCAGCACACGACAC

>V350057461L1C002R0380167231

TGGGAGTCGCCATTAAGGACCTCTGGGTATGGGAGAGCTTGAT

>V350057461L2C002R0660885580

AAAAGGTTCTTAGCAATTGTTGCCTCAGAGAGCTGCAGTGTTG

>V350057461L1C003R0470111462

AGAATCTGCAGGATGACATGGTTCGCCCACTTATCTTCCATCC

>V350057461L2C005R0270648986

TACAGGAAGGAGGTTGTGAACAGACCAGTTACGATGATGCTTG

>V350057461L1C005R0111117735

ATGACTGGGCAATTCTCGTCCTTGTGCCGAGCGAAAAGTGTTT

>V350057461L1C002R0040543810

GTTAGAGGACAGTTGAGATGTGCAAACCAGTCAAACAGAGGAT

>V350057461L1C002R0241115977

AGATATCATAGAGAAACAACGAGCCCAGAATAAGTGACCCAGT

>V350057461L2C005R0030250502

AGACGGAAGAGGAGGGGGACGTGAAGATGAATGACGAGGGTGG

>V350057461L2C005R0370381321

TCCTCTCAAGCAACTTGACCATCTCACGTATGGCTAACATGGC

>V350057461L2C004R0350587923

GAGATGAAGCAGAGAAGACTGGTCAGAGGATGCTGCTTTTCAG

>V350057461L2C005R0690658751

CCATGTGCGTGGAACTAGCAATATTACTGAGGGTATAAGGCGC

>V350057461L1C002R0650012854

GATCTTATCTTCGTCGGAATGTATATATGTATATAGACCCTCA

>V350057461L1C002R0400008605

GCTCCCACCGTCACCAAATACAAAGGGAGATTCTACTGCTTCT

>V350057461L1C006R0520408659

GACAAGAATGACCCTTTAAGCCGGGTCAACGATGCGGATGAAC

>V350057461L1C005R0720785806

ATCGTCTAAGAAAATACAATGGCTTGCAGAGAGTCGGCAAACT

>V350057461L1C005R0610324235

CGAAGACGCTGCCAATAAGGACACGCGATATCACGTGATCAAT

>V350057461L1C001R0330896078

AATTACAATCACCATCAGCCTCCATCCTCTTTTCTCCTCATCC

>V350057461L1C001R0250913828

ATCCACCAAGATAATGCAAATTTCTGACTTTGGGCTTGTTTTG

>V350057461L2C005R0460057926

CACAAGTCAGCACACTGTCCATCGCCCATGTATTTCATTGACA

>V350057461L1C004R0460393210

CTCTTCATCTCCTGGATGATACTGCAGACTGGAAGTTGGCGCT

>V350057461L2C006R0500690289

AGAGGACTGGATTTAGCTCAAAATTTTCCAGTTACCGGTTTTG

>V350057461L2C005R0340333105

GTCCAAGCTACTTAATGATTTGCGCTGGATCTTTTGTTTTCTG

>V350057461L1C002R0460663246

CTGATTAGCTATACAAATTATTAAATTATTCCTAAGAGACACG

>V350057461L1C003R0450098096

AAAGCAAATTGATATACGAAGGTTCGACACGTCCAGATGTCGG

>V350057461L1C005R0120708654

CACTTTGAGACAGTAAAGTCGCTGAATACCTGGGCTATATTCC

>V350057461L1C003R0560487068

AAAAAATCGTGGCAAGGCAGCAGTTCGTAGATGAATATTCTCT

>V350057461L2C001R0280187374

TATTCCTCCTCTTCAAGGATGTCCAGAATGAGTGTTCCCGACT

>V350057461L1C001R0631291630

GATATGATACGTTCTGACACCAACAGAGAAACCTGCGCCCTTT
